# Platelets and Antiplatelet Medication in COVID-19-Related Thrombotic Complications

**DOI:** 10.3389/fcvm.2021.802566

**Published:** 2022-01-24

**Authors:** Waltraud C. Schrottmaier, Anita Pirabe, David Pereyra, Stefan Heber, Hubert Hackl, Anna Schmuckenschlager, Laura Brunnthaler, Jonas Santol, Kerstin Kammerer, Justin Oosterlee, Erich Pawelka, Sonja M. Treiber, Abdullah O. Khan, Matthew Pugh, Marianna T. Traugott, Christian Schörgenhofer, Tamara Seitz, Mario Karolyi, Bernd Jilma, Julie Rayes, Alexander Zoufaly, Alice Assinger

**Affiliations:** ^1^Department of Vascular Biology and Thrombosis Research, Centre of Physiology and Pharmacology, Medical University of Vienna, Vienna, Austria; ^2^Division of Visceral Surgery, Department of General Surgery, Medical University of Vienna, General Hospital Vienna, Vienna, Austria; ^3^Institute of Physiology, Centre of Physiology and Pharmacology, Medical University of Vienna, Vienna, Austria; ^4^Institute of Bioinformatics, Biocenter, Medical University of Innsbruck, Innsbruck, Austria; ^5^Department of Medicine IV, Clinic Favoriten, Vienna, Austria; ^6^Institute of Cardiovascular Sciences, College of Medical and Dental Sciences, University of Birmingham, Birmingham, United Kingdom; ^7^Institute of Immunology and Immunotherapy, University of Birmingham, Birmingham, United Kingdom; ^8^Department of Clinical Pharmacology, Medical University of Vienna, General Hospital Vienna, Vienna, Austria

**Keywords:** COVID-19, infection, platelet, platelet dysfunction, microthrombi, anti-platelet therapy, thrombosis, hemostasis

## Abstract

Coronavirus disease 2019 (COVID-19) induces a hypercoagulatory state that frequently leads to thromboembolic complications. Whereas anticoagulation is associated with reduced mortality, the role of antiplatelet therapy in COVID-19 is less clear. We retrospectively analyzed the effect of anticoagulation and antiplatelet therapy in 578 hospitalized patients with COVID-19 and prospectively monitored 110 patients for circulating microthrombi and plasma markers of coagulation in the first week of admission. Moreover, we determined platelet shape change and also thrombi in postmortem lung biopsies in a subset of patients with COVID-19. We observed no association of antiplatelet therapy with COVID-19 survival. Adverse outcome in COVID-19 was associated with increased activation of the coagulation cascade, whereas circulating microthrombi did not increase in aggravated disease. This was in line with analysis of postmortem lung biopsies of patients with COVID-19, which revealed generally fibrin(ogen)-rich and platelet-low thrombi. Platelet spreading was normal in severe COVID-19 cases; however, plasma from patients with COVID-19 mediated an outcome-dependent inhibitory effect on naïve platelets. Antiplatelet medication disproportionally exacerbated this platelet impairment in plasma of patients with fatal outcome. Taken together, this study shows that unfavorable outcome in COVID-19 is associated with a profound dysregulation of the coagulation system, whereas the contribution of platelets to thrombotic complications is less clear. Adverse outcome may be associated with impaired platelet function or platelet exhaustion. In line, antiplatelet therapy was not associated with beneficial outcome.

## Introduction

Coronavirus disease 2019 (COVID-19) induced by infection with severe acute respiratory syndrome coronavirus 2 (SARS-CoV-2) is a complex syndrome primarily afflicting the airways to cause acute respiratory distress, but also causing systemic malfunction of various physiologic systems such as hemostasis (1, 2). Thromboembolic complications are common with early studies reporting overall incidences among hospitalized patients with COVID-19 ranging from 7–46%, with higher rates in critically ill patients requiring intensive care unit (ICU) treatment (3–7). More recently, several multicenter randomized controlled trials found incidences of major thrombotic events under standard prophylactic anticoagulation ranging between 2.1% in moderately ill and 10.4% in critically ill patients (8, 9); however, among patients at high risk and with very high D-dimer levels, thromboembolisms may occur in up to 29% of patients despite thromboprophylaxis (10). At the same time, 0.9–5.6% of hospitalized patients with COVID-19 experience a major or clinically relevant non-major bleeding event, with rates again being higher among the severely ill (7–9). Of note, the majority of thrombotic events are venous thromboembolisms, especially symptomatic pulmonary embolisms and deep vein thrombosis, though thrombosis may also occur at arterial sites (5, 10–12), pointing toward a complex derangement of the hemostatic system that involves both platelet function (primary hemostasis) and the coagulation cascade (secondary hemostasis).

The hypercoagulatory state induced in COVID-19 shows clinical and laboratory features that partially overlap with bacterial sepsis-induced coagulopathy (SIC) and disseminated intravascular coagulation (DIC), but represents a unique condition that has hence been termed COVID-19-associated coagulopathy (CAC) (13). CAC is characterized by widespread deregulation of coagulation parameters, such as increased D-dimer and prolonged prothrombin time, whereas platelet counts are only slightly reduced (14). The precise mechanism of CAC is still under investigation and seems highly complex due to the specific pathophysiological environment created by SARS-CoV-2 infection, which is influenced by a plethora of mediators. Immunothrombosis represents a crucial link among systemic hypercoagulability, endothelial dysfunction, and respiratory failure, where neutrophils, immunogenic platelets, and a dysregulated coagulation cascade work as partners in crime leading to immunothrombotic tissue injury in COVID-19 (15–17).

Patients with COVID-19 show increased basal platelet activation, including enhanced tissue factor and phosphatidylserine (PS) expression, augmented circulating platelet-leukocyte aggregates and raised soluble markers of platelet activation (18–21). Surface and soluble CD62P were reported to increase with COVID-19 severity and sCD62P may serve as a marker for thromboembolism (22, 23). Baseline levels of procoagulant PS-expressing platelets are also higher in patients with COVID-19 with thrombosis than in those without and elevated procoagulant platelets are associated with exacerbated sequential organ failure assessment (SOFA) score (21).

However, the impact of COVID-19 on prothrombotic and procoagulatory platelet function is multifaceted, and the underlying molecular mechanisms remain largely obscure. Although high levels of procoagulant fibrinogen-binding platelets, so-called coated platelets, are predictive of death in COVID-19, most patients exhibit lower levels of coated platelets than hospitalized SARS-CoV-2-negative controls (24). In line, despite the reported association of platelet PS expression with thrombosis and disease severity, the capacity of platelets to respond to stimulation is reduced in COVID-19 (25). Whereas some studies found that elevated basal platelet activation in severe COVID-19 translated into enhanced aggregation and prothrombotic capacity (19, 26), other studies suggested an association with platelet exhaustion (15, 20, 27, 28). These discrepancies illustrate that the role of platelet activation in COVID-19 is still exceedingly unclear, and indeed, data may be influenced by a variety of different factors ranging from virus variants, to disease stage to sampling technique.

In line, the impact of antiplatelet therapy on COVID-19 severity and outcome is currently unclear. Whereas dual antiplatelet therapy improves hypoxemia (29) and aspirin administration reduces the risk for mechanical ventilation, ICU admission and mortality without increasing the bleeding risk in some studies (30, 31), others found no protective effect of aspirin against adverse thrombotic events or mortality (32, 33).

Thus, this study aimed to provide a better understanding of the contributions of platelet dysfunction to thrombotic complications and outcome in COVID-19 using a longitudinal approach and sampling techniques optimized to study platelet dysfunction. Retrospective analysis of 578 consecutive SARS-CoV-2-positive patients uncovered no significant effect of antiplatelet therapy on cumulative survival. Whereas plasma analysis showed a clear dysregulation of endothelial quiescence and the coagulation cascade in a prospective subcohort comprising 110 patients, poor outcome was not associated with elevated circulating microthrombi. In line, pulmonary thrombi in postmortem sections were fibrin(ogen)-rich and platelet-poor, suggesting impaired platelet function. Platelets of severely ill patients with COVID-19 retained their inherent ability to form lamellipodia, which is essential for vascular integrity. However, plasma alterations in fatal disease mediated an outcome-dependent hyporesponsive platelet phenotype which was disproportionately exacerbated by antiplatelet medication.

## Methods

### Ethics Approval

Ethics approval was obtained by the local ethics committees (Medical University of Vienna: EK1315/2020 and EK1548/2020 for blood samples and EK 20-079-VK for retrospective clinical data; North East—Newcastle & North Tyneside 1 Research Ethics Committee: 19/NE/0336 for postmortem sections). Written informed consent was obtained from prospectively observed participants. The study conformed to guidelines set out by the Declaration of Helsinki and was carried out as part of the Austrian Coronavirus Adaptive Clinical Trial (ACOVACT; ClinicalTrials.gov NCT04351724).

### Retrospective Study Cohort

All consecutive patients with SARS-CoV-2 infection, confirmed by real-time PCR of naso- or oropharyngeal swab, who were admitted to the Clinic Favoriten, the primary COVID-19 hospital in Vienna, Austria, between February 2020 and February 2021, were retrospectively analyzed by retrospectively retrieving information from case report forms. All patients had available outcome data at the time of analysis. National policy demanded that all hospital patients receiving out- or inpatient treatment were tested for SARS-CoV-2 irrespective of medical complaints. Thus, our cohort also comprises patients without COVID-19-related symptoms upon admission. No virus variants were found within the study cohort. Antiplatelet therapy was defined as aspirin and/or P2Y_12_ blockers, anticoagulation was defined as low molecular weight heparin (LMWH) and/or non-vitamin-K oral anticoagulants (NOAC).

### Prospective Study Cohort

The prospective study cohort represents a subcohort of the retrospective study cohort, comprising patients who were admitted to the Clinic Favoriten between April 17, 2020 and October 28, 2020, with no significant differences in outcome distribution but less asymptomatic/mild patients. Inclusion criteria were confirmed SARS-CoV-2 infection, ≥18 years of age and study inclusion within 72 h after hospital admission. Exclusion criteria were life expectancy under one month (e.g., due to severe comorbidities), pregnancy or breastfeeding, stage 4 kidney disease, and severe liver dysfunction which could affect hemostasis.

Blood was collected every 2–3 days over 1 week following study inclusion for the assessment of circulating microthrombi by flow cytometry and of plasma components by multiplex analysis. Blood draws were not part of clinical routine and thus were skipped if a patient was anemic (hemoglobin < 11 g/dl).

### Patient Medication

Since June 2020, hospitalized patients with COVID-19 were routinely treated with prophylactic anticoagulation unless contraindicated, administering LMWH (nadroparin) at normal dosage to patients in the general ward (<70 kg/glomerular filtration rate (GFR) ≥30: 0.4 mL; <70 kg/GFR <30: 0.3 mL; >70 kg/GFR≥30: 0.6 mL; >70 kg/GFR <30: 0.4 mL) and administering high-dose prophylaxis with nadroparin or enoxaparin to ICU patients (<70 kg/GFR>30: 30 mg nadroparin 2x daily; >70 kg/GFR>30: 40 mg nadroparin 2x daily; >100 kg/GFR>30: 60 mg nadroparin 2x daily; GFR <0: 20 mg enoxaparin 2x daily).

Patients with suspected pulmonary emboli or newly occurring thromboembolic events received therapeutic anticoagulation, as was considered for patients with D-dimer >3 mg/dL. Antiplatelet therapy was administered as either 100 mg aspirin (daily), 75 mg clopidogrel (2x daily), 90 mg ticagrelor (2x daily), or a combination thereof. Long-term therapeutic anticoagulation and antiplatelet medication were continued during hospitalization.

### Patient Sample Procurement

Blood was drawn by the same personnel throughout the study into vacutainer tubes containing citrate or citrate, theophylline, adenosine, and dipyridamole (CTAD). Samples were transported immediately at room temperature to the biosafety level 2 facility of the Institute of Vascular Biology and Thrombosis Research, Medical University of Vienna (<1 h) and analyzed immediately.

### Flow Cytometric Assessment of Circulating Microthrombi

Citrate-anticoagulated whole blood was stained with α-CD42b-PerCP (HIP1, 1:75, BioLegend), α-CD66b-PacificBlue (G10F5, 1:75, BioLegend), and α-CD14-APC (MΦP9, 1:60, BD Biosciences) for 20 min, diluted with 1-step Fix/Lyse solution (eBioscience), and analyzed on a Cytoflex S cytometer using CytExpert 2.4 software (both Beckman Coulter).

Gating strategy: CD14- and CD66b-negative events with characteristic very high FSC and SSC (using a gate identifying events equal or bigger than neutrophils) were loosely defined as large events and analyzed for CD42b. CD42b-positive large events that did not fall into the singlet gate were considered circulating microthrombi (Supplementary Figure 1). Microthrombi were normalized to platelet counts to account for potential thrombocytopenia and quantified as events/ml whole blood using the calibrated flow rate and measurement time of the cytometer.

### Plasma Preparation

Plasma was prepared as previously described, ensuring minimal platelet preactivation (34). Briefly, whole blood was centrifuged for 10 min at 1.000 g at 4°C and the obtained supernatant cleared of remaining platelets and debris by high-speed centrifugation (10 min, 10.000 g). Aliquoted plasma was stored at−80°C until analysis with no additional freeze–thaw cycles.

### Multiplex Analysis

Citrate, theophylline, adenosine, and dipyridamole plasma was analyzed for soluble markers of endothelial dysfunction, thrombosis, and coagulation using predefined bead-based multiplex assay panels proinflammatory chemokines, vascular inflammation 2, thrombosis, and fibrinolysis (LegendPlex BioLegend) according to the manufacturer's instructions. Samples were measured on a Cytoflex S cytometer (Beckman Coulter) and analyzed *via* LegendPlex v8.0 software (BioLegend).

Plasma levels in ICU patients and non-survivors relative to uncomplicated cases were visualized as heatmaps showing log2-fold changes using Genesis (version 1.8.1). Differences between outcome groups were tested by two-sided Wilcoxon rank sum test using R 4.0.1 (The R Foundation for Statistical Computing, Vienna, Austria) with *p*-values adjusted for multiple testing based on the false discovery rate according to the Benjamini–Hochberg method. Differences with *p* < 0.05 were considered as statistically significant.

### *In vitro* Platelet Activation in Patient Plasmas

Citrate-anticoagulated blood from naïve healthy donors not previously exposed to SARS-CoV-2 (confirmed by IgG serology) was centrifuged for 20 min at 120 g to obtain platelet-rich plasma (PRP). Platelets were subsequently pelleted for 90 s at 1.000 g in the presence of prostacyclin (PGI_2_, 0.1 μg/ml) and resuspended in PBS at double density (500 μl PBS per ml PRP). Concentrated platelets were diluted 1:8 with patient plasma in the presence of ticagrelor (2.5 μM), heparin (2.5 U/ml), or DMSO control (10 min) before stimulation with crosslinked collagen-related peptide (CRP-XL; 50 ng/ml, 15 min; CambCol Laboratories). Platelets were stained with α-CD62P-BrilliantViolet605 (1:100) and PAC1-FITC (1:60) for 20 min before fixation in 1% formaldehyde and flow cytometric analysis. Plasma samples were obtained from matched patients with different outcomes that did not receive antiplatelet medication.

### Immunofluorescence Staining of Pulmonary Sections

Postmortem pulmonary paraffin sections from fatal COVID-19 cases were used to assess thrombosis. Demographics and laboratory findings at admission are given in Table 1. Following antigen retrieval, lung sections were treated with 3% H_2_O_2_ (15 min), blocked with PBS containing 5% bovine serum albumin (BSA) and 10% goat serum (1 h) and stained with α-CD42b, α-fibrin(ogen), and appropriate secondary antibodies conjugated to AlexaFluor647 or AlexaFluor488, respectively. Nuclei were stained using DAPI. Lung autofluorescence was quenched using a commercial kit (Vector laboratories) and slides mounted using ProLong Gold Antifade Mountant (Life Technologies). Sections were imaged using Zeiss Axio Scan.Z1 microscope. Individual thrombi were classified into mid-sized thrombi (≥500 μm maximal diameter) and microthrombi (<500 μm) and evaluated for the platelet content. Pictures of individual thrombi were evaluated using CellProfiler for CD42b- and fibrin(ogen)-positive area.

**Table 1:** Patient demographics (postmortem lung sections).

	**Missing data**	**All (*n* = 8)**
**Parameter**	***n***	***n* (%)**
		**Median (IQR)**
**Sex**	–	
Male		3 (37.5)
Female		5 (62.5)
**Age (years)**	–	74 (67–86.5)
**Comorbidities**		
Obesity (BMI > 25)	–	5 (62.5)
Diabetes type II	1	2 (28.6)
Hypertension	–	3 (37.5)
Coronary heart disease	–	1 (12.5)
Chronic obstructive pulmonary disease	1	2 (28.6)
Asthma	–	1 (12.5)
Malignancy	–	2 (25.0)
**Antithrombotic medication** ^§^		
Antiplatelet therapy	–	3 (37.5)
Anticoagulation therapy	1	0 (0.0)
**Clinical Characteristics**		
Admitted to hospital	–	5 (62.5)
Total hospitalization (days)	–	17 (7–22)
Invasive ventilation	–	1 (12.5)
**Postmortem pulmonary pathologies**		
Thrombosis in arteries/arterioles	–	4 (50.0)
Pulmonary embolism	–	0 (0.0)
Edema	–	4 (50.0)

§ *Antiplatelet therapy comprises aspirin and/or P2Y_12_ blockers, anticoagulation comprises LMWH and/or NOAC; BMI, body mass index; IQR, interquartile range*.

### Platelet Spreading

Channel μ-slides VI0.4 (ibidi) were coated with fibrinogen (100 μg/ml, 1 h at 37°C), washed with PBS and water, and left to dry. Washed platelets from healthy naïve donors or patients with COVID-19 requiring ICU treatment were prepared as described above. Platelets were resuspended in PBS at 7% density of PRP (1.4 ml PBS per 100 μl PRP), seeded into channel slides, and allowed to attach and spread for 30 min before fixation with 1% formaldehyde. Platelets were permeabilized with 0.5% Triton-X100 (4 min), blocked with 10%FBS/1% BSA (1 h), and stained with α-CD42b-AlexaFluor488 (1:50) over night at 4°C before mounting in PBS/glycerol (1:6). Pictures of at least 100 platelets per donor were taken at 100 x magnification using Nikon A1 plus confocal laser-scanning microscope and NIS software. Platelet shape of individual platelets was classified into four categories using a CellProfiler pipeline: discoid, filopodia-forming, lamellipodia-forming and fully spread.

### Statistics and Data Presentation

Statistical testing and graph preparation were done with IBM SPSS 27, GraphPad Prism 8, and SankeyMATIC online tool. Gaussian distribution of metric data was tested by Kolmogorov–Smirnov and Shapiro–Wilk test. Accordingly, differences in plasma components of ICU patients or non-survivors relative to uncomplicated cases upon study entry were tested by Wilcoxon rank sum test, and differences over time were tested by a mixed-effects model with Geisser-Greenhouse correction. Two-way ANOVA was used to test differences in platelet composition between different sized thrombi and to test differences in platelet spreading stages between healthy donors and patients with COVID-19. Differences in platelet activation between control- and ticagrelor/heparin-treated platelets were tested by repeated measures two-way ANOVA, and outcome-specific effects of ticagrelor/heparin were tested by repeated measures 3-way ANOVA. Differences between nominal data were assessed by chi-square test, whereas differences in cumulative survival were examined by age-adjusted Cox regression analysis. Time lines show median values with interquartile range, and violin plots show median (line) and quartiles (dotted line).

## Results

### Antiplatelet Medication Is Not Associated With Altered Survival of Hospitalized Patients With COVID-19

**Figure 1:**
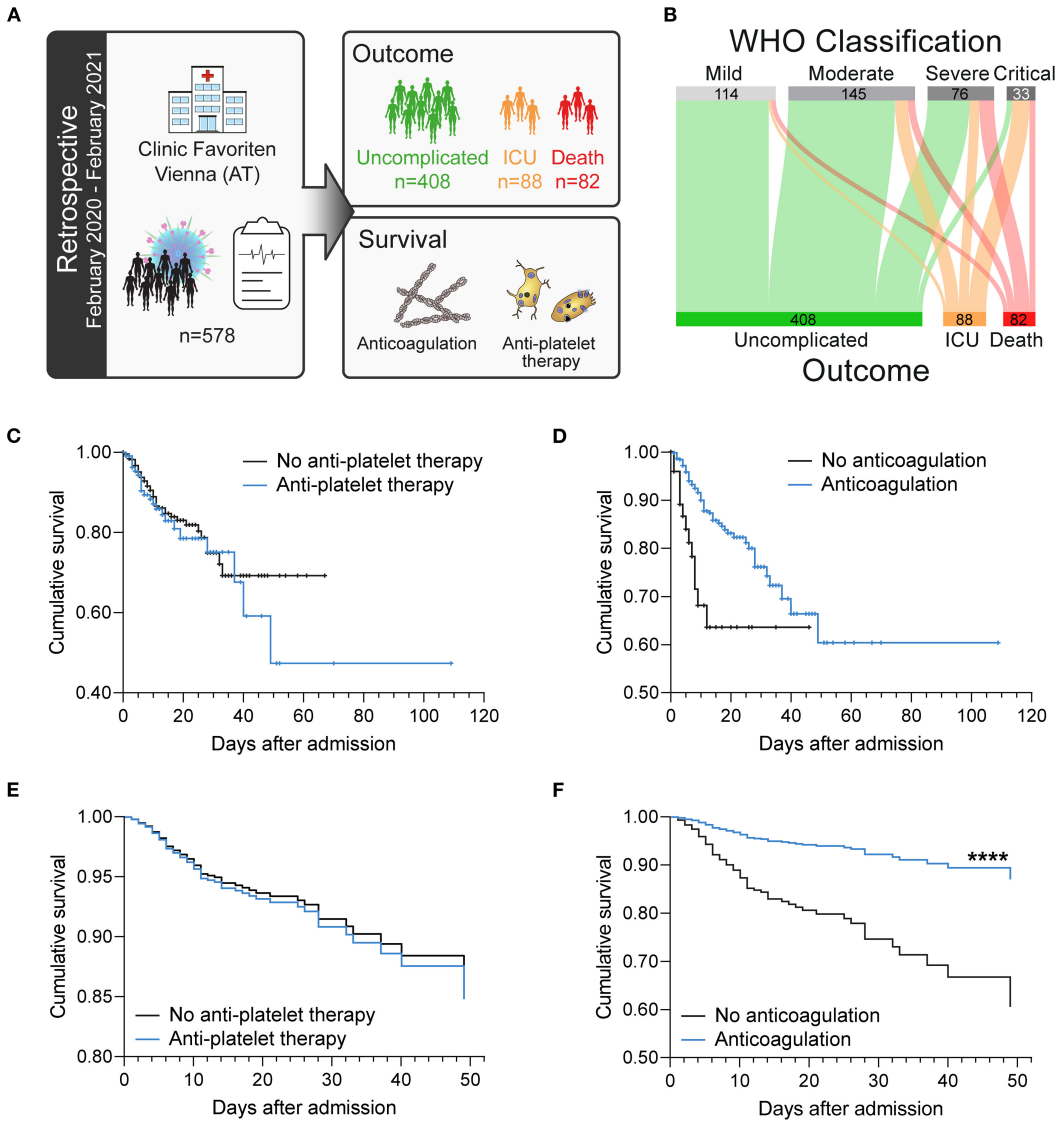
Antiplatelet therapy does not affect the survival of patients with COVID-19 in a retrospective analysis after adjusting for age. **(A)** Retrospective study design: 578 consecutive patients with confirmed SARS-CoV-2 infection admitted to the primary COVID-19 hospital in Vienna, Austria were included in this longitudinal study and retrospectively analyzed. **(B)** Severity classification according to WHO guidelines is not stringent for fatal outcome. **(C–F)** Effect of **(C,E)** antiplatelet therapy (aspirin and/or P2Y_12_ blockers) or **(D,F)** anticoagulation (LMWH and/or NOAC) on survival. **(C,D)** Kaplan–Meier curves. **(E,F)** Age-adjusted COX regression analysis. ^****^p < 0.0001. *n* = 578 patients.

We retrospectively analyzed 578 consecutive patients with SARS-CoV-2 infection who were hospitalized at the primary COVID-19 hospital in Vienna, Austria (Clinic Favoriten), between February 2020 and February 2021 (Figure 1A).

Patients were classified upon admission into disease severity classes according to the World Health Organization (WHO) classification. However, although higher WHO classification at admission was generally associated with worse course of disease, 4% of patients classified as mild still died, whereas 18% of patients classified as critical showed uncomplicated course of disease without the need for intensive care (Table 2). We therefore stratified our cohort according to outcome into patients with uncomplicated course of disease (*n* = 408; 70.6%), patients requiring ICU treatment (*n* = 88; 15.2%) and patients who died (*n* = 82; 14.2%) (Figure 1B; Table 2).

**Table 2:** Patient demographics (retrospective cohort).

	**Missing data**	**All** **(*n* = 578)**	**Uncomplicated** **(*n* = 408; 70.6%)**	**ICU** **(*n* = 88; 15.2%)**	**Death** **(*n* = 82; 14.2%)**	
**Parameter**	***n***	***n* (%)** **Median (IQR)**	***n* (%)** **Median (IQR)**	***n* (%)** **Median (IQR)**	***n* (%)** **Median (IQR)**	*p*-value^*^
**Sex**	–					***p*** **=** **0.021**
Male		235 (40.7)	175 (42.9)	24 (27.3)	36 (43.9)	
Female		343 (59.3)	233 (57.1)	64 (72.7)	46 (56.1)	
**Age (years)**	6	60 (46–75)	56 (43–71)	58.5 (50–70)	80 (74–89)	***p*** **<** **0.001**
**Comorbidities**						
Current smoker	173	38 (9.4)	25 (8.3)	8 (11.4)	5 (14.3)	*p* = 0.423
Obesity (BMI > 25)	54	162 (30.9)	112 (30.1)	36 (41.4)	14 (21.5)	***p*** **=** **0.027**
Diabetes type II	8	144 (25.3)	89 (22.2)	30 (34.1)	25 (30.9)	***p*** **=** **0.031**
Hypertension	6	285 (49.8)	179 (44.5)	51 (58.0)	55 (67.1)	***p*** **<** **0.001**
Cardiovascular disease (any)	8	141 (24.7)	80 (20.0)	19 (21.6)	42 (51.9)	***p*** **<** **0.000**
Coronary heart disease	6	77 (13.5)	44 (10.9)	14 (15.9)	19 (23.5)	***p*** **=** **0.008**
Chronic heart failure	7	33 (5.8)	16 (4.0)	3 (3.4)	14 (17.3)	***p*** **<** **0.001**
Atrial fibrillation	7	73 (12.8)	38 (9.4)	8 (9.1)	27 (33.8)	***p*** **<** **0.001**
Peripheral arterial disease	7	25 (4.4)	11 (2.7)	2 (2.3)	12 (14.8)	***p*** **<** **0.001**
Chronic obstructive pulmonary disease	6	43 (7.5)	20 (5.0)	7 (8.0)	16 (19.8)	***p*** **<** **0.001**
Asthma	6	20 (3.5)	15 (3.7)	3 (3.4)	2 (2.5)	*p* = 0.854
Hypo-/Hyperthyroidism	7	69 (12.1)	44 (10.9)	10 (11.4)	15 (18.5)	*p* = 0.158
Chronic renal insufficiency	6	76 (13.3)	42 (10.4)	6 (6.8)	28 (34.6)	***p*** **<** **0.001**
Chronic liver disease	7	26 (4.6)	19 (4.7)	3 (3.4)	4 (5.0)	*p* = 0.850
Malignancy	6	31 (5.4)	18 (4.5)	4 (4.5)	9 (11.1)	*p* = 0.051
**Antithrombotic medication** ^§^						
Antiplatelet therapy	27	114 (20.3)	66 (16.7)	26 (29.9)	22 (28.2)	***p*** **=** **0.004**
Anticoagulation therapy	15	522 (92.7)	368 (92.9)	85 (96.6)	69 (87.3)	*p* = 0.068
**COVID-19 classification at admission** ^†^	210	368 (63.7)				***p*** **<** **0.001**
Asymptomatic/mild		114 (31.0)	106 (37.6)	3 (6.1)	5 (13.5)	
Moderate		145 (39.4)	122 (43.3)	15 (30.6)	8 (21.6)	
Severe		76 (20.7)	48 (17)	11 (22.4)	17 (45.9)	
Critical		33 (9.0)	6 (2.1)	20 (40.8)	7 (18.9)	
**Clinical Characteristics**						
Total hospitalization (days)	6	10 (6–16)	9 (6–14)	19 (13–30)	7.50 (4–12.25)	***p*** **<** **0.001**
Invasive ventilation	8	48 (8.4)	0 (0)	32 (37.6)	16 (19.8)	***p*** **<** **0.001**
**Prospective Analysis**		110	73 (66.4)	26 (23.6)	**11 (10.0)**	***p*** **=** **0.016**

* *p < 0.05 considered significant (bold). Nominal variables were compared by chi-square test, metric variables by Kruskal–Wallis test;*

§ *Antiplatelet therapy comprises aspirin and/or P2Y_12_ blockers, anticoagulation comprises LMWH and/or NOAC;*

† *COVID-19 classification according to the guidelines issued by the World Health Organization in mild (fever <38°C, no dyspnea, no pneumonia), moderate (fever, respiratory symptoms, pneumonia), severe (respiratory distress with respiratory rate ≥ 30 per min, oxygen saturation <93% at rest) and critical (respiratory failure with requirement of mechanical ventilation, requirement of ICU); BMI, body mass index; ICU, intensive care unit; IQR, interquartile range*.

Outcome was associated with differences in age, sex, and the presence of comorbidities of the metabolic and cardiovascular system and also chronic obstructive pulmonary disease (COPD) and chronic renal insufficiency (CRI). Patients requiring ICU treatment displayed higher frequency of female sex, obesity, and diabetes type II, whereas non-survivors were older and showed higher frequency of cardiovascular diseases, COPD, and CRI (Table 2). Complicated disease (ICU, death) was further associated with a dysregulation of coagulation parameters (D-dimer, prothrombin time, international normalized ratio, activated partial thromboplastin time), but not with altered platelet counts (Tables 3A,B).

**Table 3A:** Laboratory findings at admission (retrospective cohort).

	**Missing data**	**All** **(*n* = 578)**	**Uncomplicated** **(*n* = 408; 70.6%)**	**ICU** **(*n* = 88; 15.2%)**	**Death** **(*n* = 82; 14.2%)**
**Parameter**	***n***	**Median (IQR)**	**Median (IQR)**	**Median (IQR)**	**Median (IQR)**
Hemoglobin (g/dL)	145	13.2 (11.8–14.4)	13.3 (12.0–14.5)	13.5 (12.5–14.4)	11.6 (10.5–13.2)
Red blood cell count (×10^12^/L)	144	4.6 (4.1–5–0)	4.6 (4.2–5.1)	4.6 (4.2–5.1)	4.2 (3.5–4.6)
Platelet count (×10^9^/L)	142	193 (152–242)	196 (154–244)	193 (142–220)	181.5 (134–247.5)
Leukocyte count (×10^9^/L)	141	5.6 (4.3–7.7)	5.5 (4.1–7.4)	6.2 (5.0–8.3)	6.1 (4.6–11.0)
Lymphocyte count (×10^9^/L)	178	1.0 (0.7–1.3)	1.1 (0.7–1.4)	0.9 (0.7–1.1)	0.8 (0.6–1.0)
Neutrophil count (×10^9^/L)	169	4.1 (3.0–5.9)	3.9 (2.7–5.5)	4.9 (3.9–8.0)	4.5 (3.3–7.9)
Monocyte count (×10^9^/L)	181	0.4 (0.3–0.5)	0.4 (0.3–0.5)	0.3 (0.2–0.5)	0.4 (0.3–0.6)
Eosinophil count (×10^9^/L)	179	0.01 (0.00–0.05)	0.02 (0.01–0.06)	0.01 (0.00–0.02)	0.01 (0.01–0.02)
Basophil count (×10^9^/L)	182	0.02 (0.01–0.04)	0.02 (0.01–0.04)	0.02 (0.01–0.04)	0.02 (0.01–0.03)
C-reactive protein (mg/L)	154	42.8 (16.5–82.6)	32.5 (10.3–73.0)	66.7 (29.6–111.6)	63.7 (31.4–104.5)
D-dimer (mg/L)	178	0.8 (0.5–2.4)	0.7 (0.4–1.9)	0.9 (0.6–1.7)	2.0 (1.0–4.2)
Prothrombin time (%)	173	101.6 (88.0–111.4)	102.9 (90.0–112.2)	104.7 (90.3–111.3)	89.1 (69.0–98.2)
International normalized ratio	174	1.0 (1.0–1.1)	1.0 (1.0–1.1)	1.0 (1.0–1.0)	1.1 (1.0–1.3)
Activated partial thromboplastin time (s)	185	30.6 (27.8–34.4)	30.1 (27.5–33–5)	31.3 (28.5–36.0)	33.5 (30.2–40.8)

*ICU, intensive care unit; IQR, interquartile range*.

**Table 3B:** Statistical analysis of laboratory findings at admission (retrospective cohort).

		**Bonferroni correction** ^+^		
**Parameter**	*p*-value^*^	**Uncomplicated - ICU**	**ICU - death**	**Uncomplicated - death**
Hemoglobin (g/dL)	***p*** **<** **0.001**	*p* = 1.000	***p*** **<** **0.001**	***p*** **<** **0.001**
Red blood cell count (×10^12^/L)	***p*** **<** **0.001**	*p* = 1.000	***p*** **<** **0.001**	***p*** **<** **0.001**
Platelet count (×10^9^/L)	*p* = 0.128			
Leukocyte count (×10^9^/L)	***p*** **=** **0.004**	***p*** **=** **0.044**	*p* = 1.000	***p*** **=** **0.021**
Lymphocyte count (×10^9^/L)	***p*** **<** **0.001**	***p*** **=** **0.003**	*p* = 1.000	***p*** **=** **0.001**
Neutrophil count (×10^9^/L)	***p*** **<** **0.001**	***p*** **<** **0.001**	*p* = 1.000	***p*** **=** **0.008**
Monocyte count (×10^9^/L)	***p*** **<** **0.001**	*p* = 0.125	***p*** **=** **0.022**	*p* = 0.421
Eosinophil count (×10^9^/L)	***p*** **=** **0.006**	***p*** **=** **0.007**	*p* = 1.000	*p* = 0.444
Basophil count (×10^9^/L)	***p*** **=** **0.095**			
C-reactive protein (mg/L)	***p*** **<** **0.001**	***p*** **<** **0.001**	*p* = 1.000	***p*** **=** **0.001**
D-dimer (mg/L)	***p*** **<** **0.001**	*p* = 0.283	***p*** **=** **0.007**	***p*** **<** **0.001**
Prothrombin time (%)	***p*** **<** **0.001**	*p* = 1.000	***p*** **<** **0.001**	***p*** **<** **0.001**
International normalized ratio	***p*** **<** **0.001**	*p* = 1.000	***p*** **<** **0.001**	***p*** **<** **0.001**
Activated partial thromboplastin time (s)	***p*** **<** **0.001**	*p* = 0.102	*p* = 0.222	***p*** **<** **0.001**

* *Kruskal–Wallis test for 3 independent groups (asymptotic significance, not adjusted);*

+ *Kruskal–Wallis 1-way ANOVA with Bonferroni correction for multiple testing (adjusted significance); p < 0.05 considered significant (bold). ICU, intensive care unit*.

Analysis of patient's survival using Kaplan–Meier curves showed no association of antiplatelet therapy (aspirin and/or P2Y_12_ receptor antagonists) with survival (Figure 1C), whereas anticoagulation (LMWH and/or NOAC) was associated with increased survival (Figure 1D). Notably, more patients with complicated course of disease (ICU, death) were elder and on antiplatelet therapy due to cardiovascular comorbidities (Table 2).

After age-adjustment, we could not observe any significant effect of antiplatelet therapy on survival (Figure 1E), whereas anticoagulation therapy was associated with ameliorated survival (Figure 1F) in a Cox regression analysis.

### Fatal Disease Is Associated With Disturbances of the Coagulation System and Vascular Dysfunction

**Figure 2:**
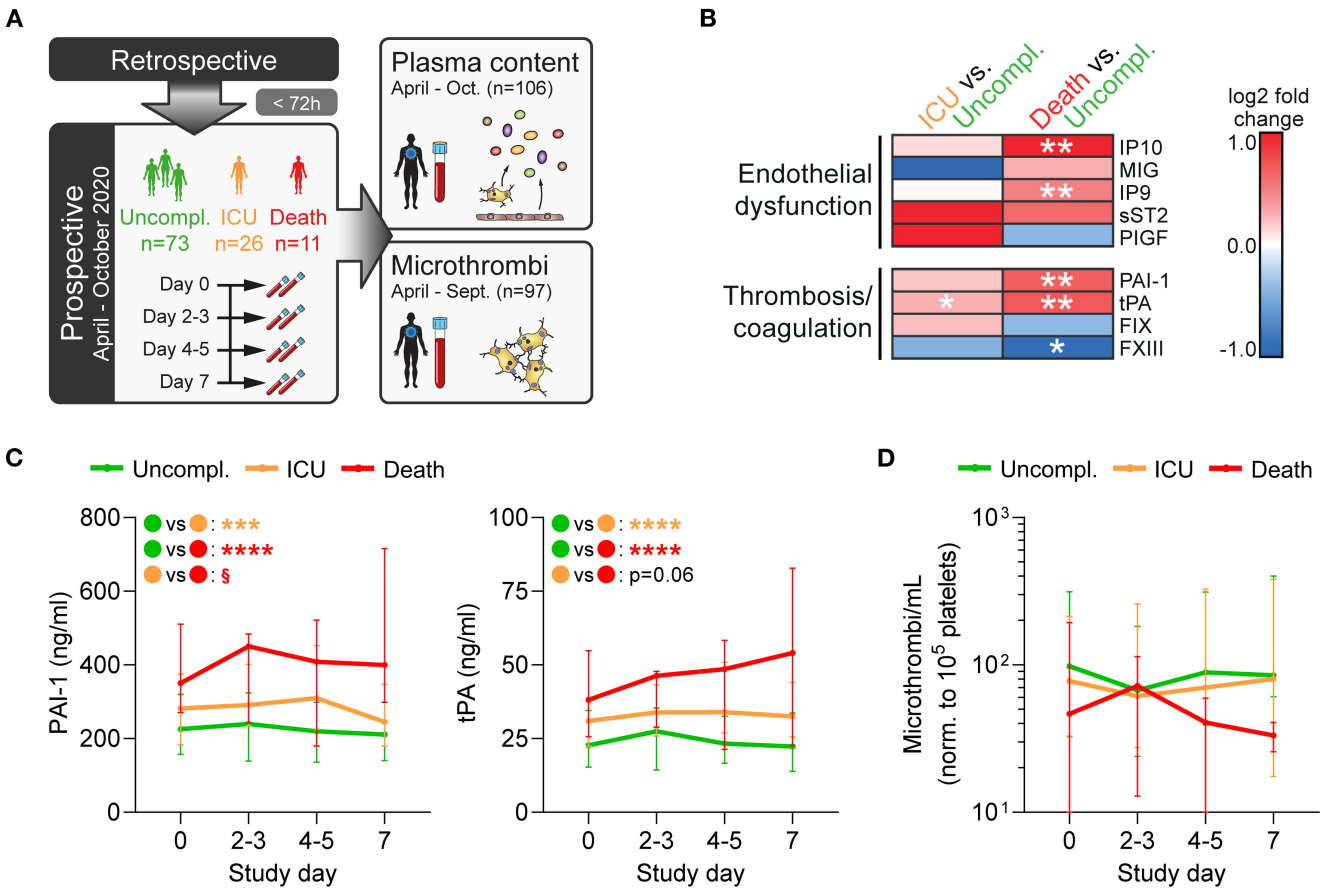
Fatal disease is associated with disturbances of the coagulation system and vascular dysfunction. **(A)** Prospective study design: In a subcohort of the retrospective cohort comprising 110 patients (enrolled within 72 h after hospital admission), blood was collected every 2–3 days over 1 week to assess plasma components and circulating microthrombi. **(B,C)** Plasma was analyzed by multiplex analysis for factors involved in endothelial dysfunction or thrombosis/coagulation. **(B)** Heatmap shows log2-fold changes in expression profiles of ICU patients or non-survivors relative to uncomplicated cases in plasma obtained at day 0. Differences were tested using two-sided Wilcoxon rank sum test. **(C)** Plasma levels of PAI-1 (left) and tPA (right) were monitored over 1 week after study entry using bead-based multiplex assay. Differences between outcome groups were tested by mixed-effects model with Geisser-Greenhouse correction. Asterisks (*) indicate significant differences to uncomplicated, section signs (^§^) indicate significant differences between ICU and death. *n* = 106 patients. **(D)** Circulating microthrombi in whole blood were monitored over 1 week after study entry by flow cytometry. *n* = 97 patients. *p < 0.05, **p < 0.01, ***p < 0.001, ****p < 0.0001; ^§^*p* < 0.001.

As immunothrombosis and coagulopathy represent crucial contributors to COVID-19 pathogenesis (14, 15), we prospectively analyzed a subcohort of 110 patients with COVID-19 admitted to Clinic Favoriten between April and October 2020 and monitored circulating markers of hemostatic dysregulation over the first week of their hospital stay. We measured a panel of plasma factors involved in vascular function, thrombosis, and coagulation and also circulating microthrombi and assessed their association with disease outcome (Figure 2A).

Fatally ill patients had significantly increased levels of interferon-γ inducible protein 9 (IP9/CXCL11) and IP10 (CXCL10), whereas no significant outcome-specific changes were observed for monokine induced by γ-interferon (MIG/CXCL9), soluble IL-33 receptor (sST2), and placental growth factor (PlGF) (Figure 2B). Additionally, ICU requirement and fatal outcome were associated with elevated tissue plasminogen activator (tPA) and plasminogen activator inhibitor 1 (PAI-1), whereas coagulation factor XIII (FXIII) levels that primarily derive from platelets were reduced (Figures 2B,C), underlining the dysregulation of the coagulation system.

Interestingly, patients with fatal disease displayed slightly lower numbers of circulating microthrombi at study entry and also during the following week, though differences were not significant (Figure 2D). These results further confirm that whereas secondary hemostasis is activated in COVID-19, the contribution of primary hemostasis is less clear which may explain the lack of benefit of antiplatelet therapy.

### Pulmonary Thrombi in Fatal COVID-19 Are Low in Platelets

**Figure 3:**
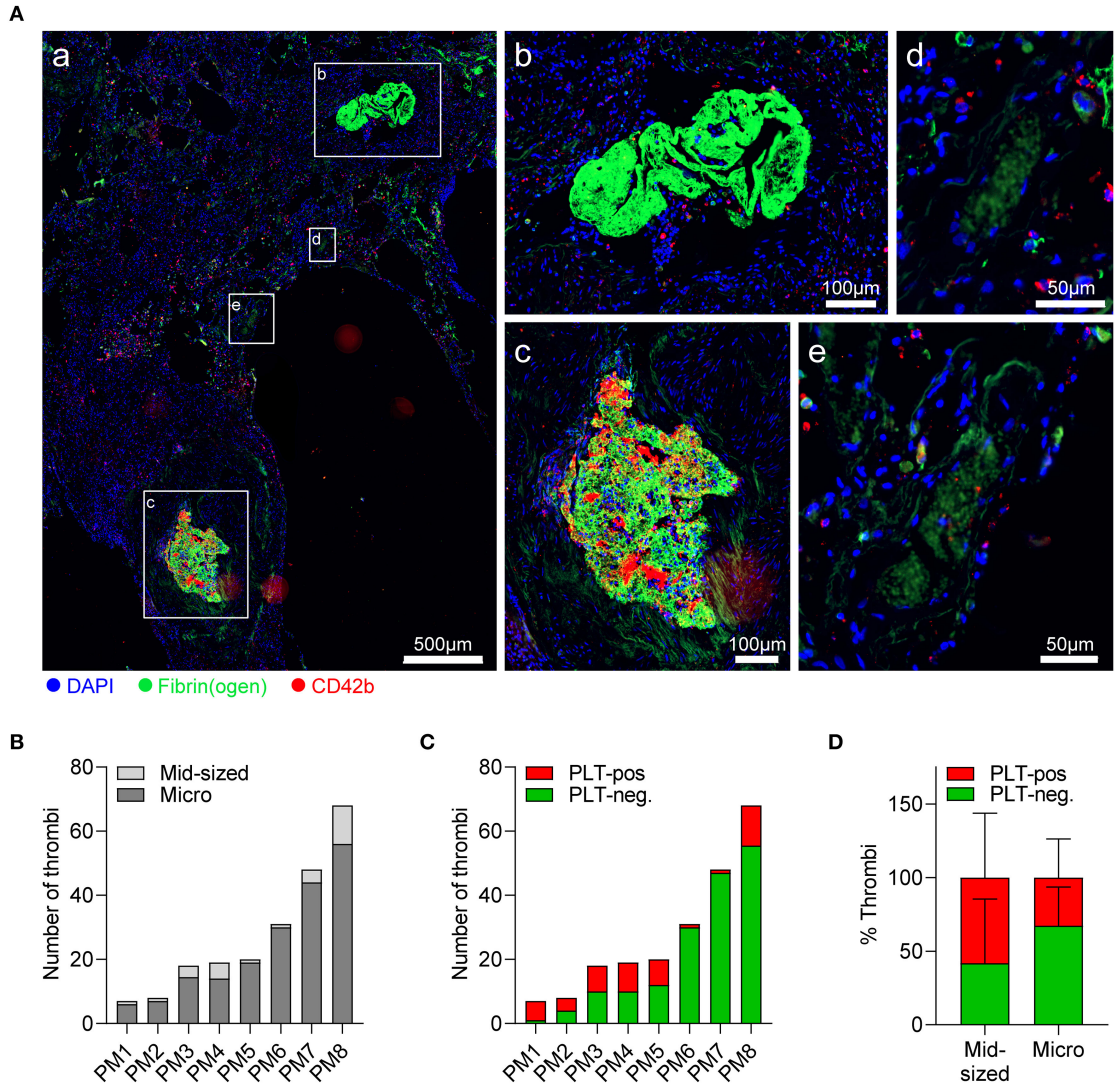
Pulmonary thrombi in COVID-19 are platelet-poor but platelets from severe COVID-19 cases show normal spreading on fibrinogen. Postmortem lung biopsies of 8 patients with COVID-19 were stained for CD42b (red), fibrin(ogen) (green), and DAPI (blue). **(A)** Representative (a) overview of lung sections showing (b) platelet-negative and (c) platelet-positive mid-sized thrombi (≥500 μm), and (d) platelet-negative and (e) platelet-positive microthrombi (<500 μm). **(B,C)** Thrombi in individual sections were stratified according to **(B)** size into mid-sized thrombi (≥500 μm) and microthrombi (<500 μm) or **(C)** according to platelet content into platelet-positive or platelet-negative. **(D)** Percentage of platelet-negative and platelet-positive thrombi among mid-sized and microthrombi. Differences in platelet composition between mid-sized and microthrombi were tested by two-way ANOVA. *n* = 8 patients.

To get a clearer picture of thrombotic complications in fatal COVID-19, we examined pulmonary postmortem biopsies of COVID-19 non-survivors for evidence of hemostatic dysregulation. Whereas sections revealed considerable heterogeneity among patients, the vast majority of thrombi were microthrombi (<500 μm) (Figures 3A,B; Supplementary Figure 2A), and only 50% of cases displayed clinically relevant thrombi in larger arteries or arterioles (Table 1). At the same time, edema was detected in 50% of lung biopsies (Table 1), indicating compromised vascular integrity. Thrombus platelet content was highly varied between patients (Figure 3C; Supplementary Figure 2B). A large fraction of mid-sized (≥500 μm) and particularly microthrombi only contained fibrin(ogen) but no platelets, which may reflect involvement of secondary but not primary hemostasis (Figure 3D). Accordingly, even when thrombi contained platelets (platelet-positive thrombi), only few scattered CD42b-positive events were observed (Figure 3A,c,e).

Evaluation of individual thrombi revealed ring-like platelet arrangements surrounding multiple microthrombi (Supplementary Figure 2C), which we speculated to reflect platelet accumulation and adhesion to leaky vessels. To assess whether reduced platelet content in thrombi was associated with diminished platelet–vessel wall interactions, we measured CD42b/fibrin(ogen) ratios in pictures of single thrombi. However, CD42b/fibrin(ogen) ratio was comparable between thrombi independently of size or platelet content (Supplementary Figures 2D,E), suggesting that there was no association between thrombi size and platelet-mediated vascular surveillance.

### Platelet Dysfunction, Vascular Integrity, and Hemostatic Complications in COVID-19

**Figure 4:**
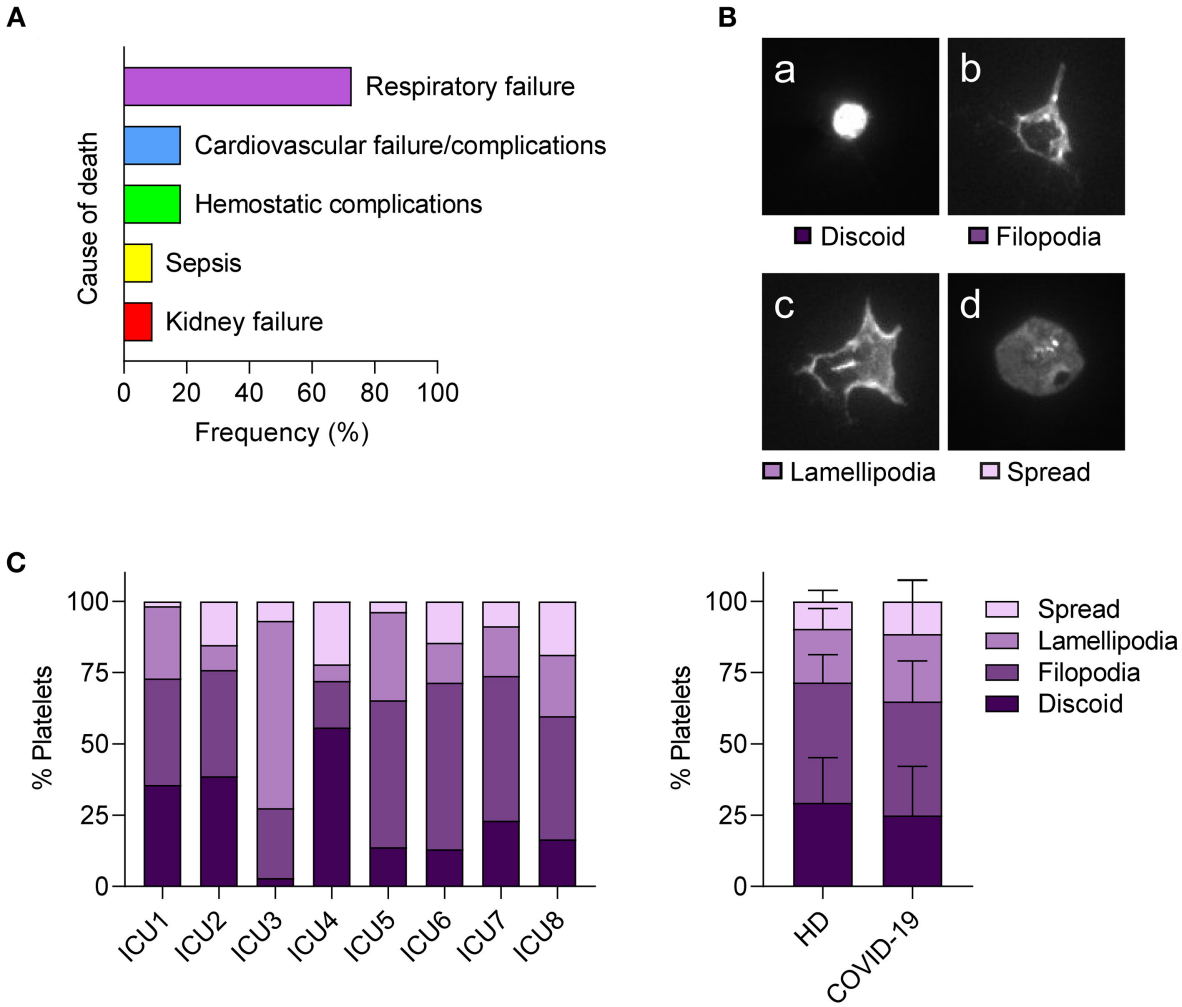
Hemostatic complications are common in fatal COVID-19 but platelets from severe COVID-19 cases show normal spreading. **(A)** Relative frequency of listed cause of death among fatal cases within the prospective study cohort. *n* = 11 patients. **(B,C)** Platelets of patients with COVID-19 requiring ICU or healthy controls were seeded on fibrinogen (30 min) and platelet spreading evaluated by immunofluorescence. **(B)** Platelets were classified into four stages: (a) discoid, (b) filopodia-forming, (c) lamellipodia-forming, and (d) fully spread. **(C)** Relative abundance of platelet spreading stages of individual patients with COVID-19 (left panel). Mean percentages of platelets in different spreading stages were compared between healthy donors (HD) and patients with COVID-19 (right panel). Differences between HD and patients with COVID-19 were tested by two-way ANOVA. *N* = 7 healthy donors/8 patients.

Based on the high prevalence of edema and the platelet-poor thrombus composition in postmortem lung biopsies, we speculated that platelet dysregulation in COVID-19 may affect their vascular surveillance function rather than thrombosis. Of note, in our prospective patient cohort, 18% of deceased patients suffered from hemostatic complications such as (pulmonary) bleeding as causes of death (Figure 4A; Supplementary Figure 3).

To examine the capacity of platelets to maintain vessel integrity in COVID-19 in more detail, we performed an *in vitro* settlement assay and compared platelet shape change in patients with COVID-19 requiring ICU treatment and healthy controls by classifying adherent platelets into four stages (discoid, filopodia-forming, lamellipodia-forming, and fully spread) (Figure 4B). We found strong variations between patients with COVID-19 and no significant differences in spreading phenotypes compared to healthy controls (Figure 4C), indicating that platelets from patients with COVID-19 do not show spreading abnormalities. In particular, lamellipodia formation, which is essential for maintaining vascular integrity but not for thrombus formation (35), was similar between patients with COVID-19 and healthy controls.

### Antiplatelet Therapy Exacerbates Plasma-Mediated Platelet Dysregulation in Patients With Adverse Outcome

**Figure 5:**
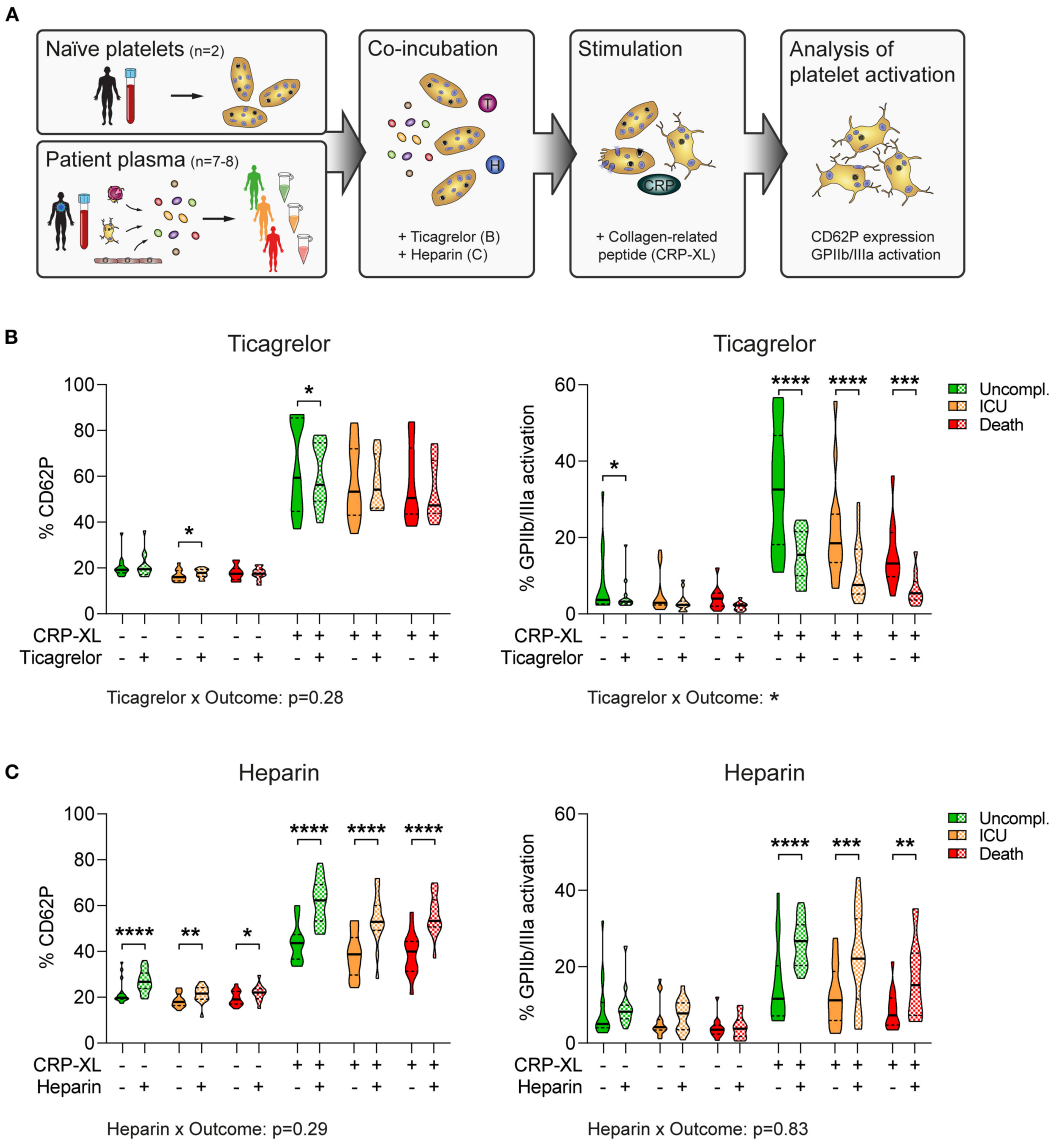
Plasma-mediated reduced platelet GPIIb/IIIa activation is disproportionately exacerbated by antiplatelet therapy in fatal COVID-19. **(A)** Experimental setup: Naïve platelets of healthy donors were incubated with plasma of patients with COVID-19 with different outcomes in the presence of ticagrelor or heparin, followed by stimulation with CRP-XL and evaluation of platelet activation *via* flow cytometry. **(B,C)** Naïve platelets were incubated in patient plasma in the presence of DMSO (solid), **(B)** 2.5 μM ticagrelor, or **(C)** 2.5 U/ml heparin (both checkered) for 10 min before stimulation with CRP-XL (50 ng/ml, 15 min) and measurement of CD62P expression (left panel) and GPIIb/IIIa activation (right panel). Differences between DMSO control and ticagrelor/heparin were tested by two-way ANOVA. Outcome-specific effects of ticagrelor or heparin were evaluated 3-way ANOVA. *n* = 2 platelet donors/7–8 patient plasmas per outcome. *p < 0.05, **p < 0.01, ***p < 0.001, ****p < 0.0001.

Since we observed low platelet content in pulmonary thrombi, but did not detect any inherent dysregulation of isolated platelets themselves, we investigated whether plasma mediated any effects on platelet function in COVID-19. Further, as patients with COVID-19 are often routinely treated with anticoagulants and/or antiplatelet medication to curtail thrombotic complications, we investigated how these medications impacted on plasma-mediated effects on platelets (Figure 5A).

We incubated naïve platelets with patient's plasma in the presence of ticagrelor or heparin before assessing platelet reactivity in response to the GPVI agonist collagen-related peptide (CRP-XL). Of note, plasma donors were void of antiplatelet medication, whereas all patients received anticoagulation. Whereas platelet CD62P induction was independent of plasma donor's outcome (*p* = 0.19 for DMSO; *p* = 0.18 for ticagrelor), GPIIb/IIIa activation was higher in plasma of uncomplicated cases than in plasma of patients with adverse outcome (*p* < 0.001 for DMSO; *p* < 0.01 for ticagrelor) (Figure 5B).

Moreover, ticagrelor had only slight inhibitory effects on CD62P expression, but substantially impaired GPIIb/IIIa activation (Figure 5B), which was in line with previous reports showing that GPIIb/IIIa activation is highly dependent on feedback mechanisms *via* ADP/P2Y_12_ (36). Accordingly, we could not detect any interaction between outcome and ticagrelor for CD62P expression (*p* = 0.28). In contrast, ticagrelor had distinct effects on GPIIb/IIIa activation in dependence of the plasma donor outcome (*p* < 0.05), leading to stronger relative platelet inhibition in plasma of non-survivors (Figure 5B). Contrarily, heparin enhanced platelet activation *in vitro*, augmenting both CD62P expression and GPIIb/IIIa activation independently of outcome (CD62P: *p* = 0.29; GPIIb/IIIa: *p* = 0.83) (Figure 5C).

Thus, plasma components appear to confer a hyporeactive platelet phenotype in severe COVID-19 cases, which may be exacerbated by antiplatelet therapy particularly in non-survivors.

## Discussion

In this study, we retrospectively analyzed the impact of antiplatelet therapy on the survival of hospitalized patients with COVID-19 and prospectively monitored plasma markers, circulating microthrombi, and platelet spreading to determine potential effects of platelet dysregulation on hemostatic complications and patient outcome. We found that anticoagulation but not antiplatelet therapy was associated with ameliorated survival in COVID-19 and that complicated disease was associated with enhanced endothelial activation and coagulation dysregulation, whereas platelet-derived microthrombi did not increase in patients with adverse outcome. In line, postmortem analysis of lung sections showed fibrinogen-rich but platelet-low thrombi. Whereas we found platelet spreading to be normal in patients with COVID-19, *in vitro* experiments revealed that plasma of non-survivors mediated an inhibitory effect on naïve platelets relative to uncomplicated cases that was disproportionately exacerbated by antiplatelet medication.

COVID-19 is often accompanied by a dysregulation of the hemostatic system with distinct features known as CAC. Hallmark characteristics of CAC are increased D-dimer and prolongation of prothrombin time with concomitant reductions in platelet count (14), pointing toward involvement in both primary and secondary hemostasis. Earlier studies on the use of prophylactic and/or therapeutic anticoagulation in COVID-19 found that anticoagulation was associated with improved survival irrespective of dosage (37, 38) and prophylactic treatment with LMWH was quickly included into standard treatment of hospitalized patients. In-depth analysis of our retrospective cohort revealed similarly beneficial association of LMWH treatment with survival, which may also be supported by antiviral effects of LMWH (39). However, the effect of antiplatelet therapy on patient's survival is less clear.

Previously, aspirin has been associated with reduced need for mechanical ventilation, ICU treatment, and decreased in-hospital mortality of patients with COVID-19 (30). However, an earlier propensity-matched study found no protective effect of aspirin on thrombosis or death in COVID-19 (32). To unravel the contribution of primary and secondary hemostasis to adverse effects in COVID-19, we analyzed the association of anticoagulation (LMWH and/or NOAC) or antiplatelet therapy (aspirin and/or P2Y_12_ blockers) with patient survival in a retrospective cohort comprising 578 consecutive patients with COVID-19. In contrast to anticoagulation which was associated with improved survival, antiplatelet therapy had no effect on the survival in our cohort after adjusting for age. This observation is in line with an ongoing large randomized controlled trial comprising almost 15,000 patients which reported no link between aspirin and mortality or risk for invasive ventilation (33).

The fact that despite high incidence of thromboembolic complications in ICU patient and the occurrence of inflammatory microthrombi in various organs of fatal COVID-19 cases, antiplatelet medication has no beneficial effect (5, 15, 40) suggests that thromboembolic complications in COVID-19 may be independent of classical prothrombotic platelet functions.

To unravel the contribution of primary and secondary hemostasis for patient outcome in COVID-19, we monitored circulating mediators and microthrombi in blood of patients with different disease courses. Conforming to the proinflammatory environment induced by SARS-CoV-2, endothelial dysfunction increased as patient outcome worsened. In particular, fatal disease was characterized by upregulated levels of IP9/CXCL11 and IP10/CXCL10, which are also expressed by microvascular endothelial cells upon activation or infection with dengue virus (41, 42), suggesting a general rather than SARS-CoV-2-specific response to viral infection. IP9 and IP10 were also found to be elevated in certain types of interstitial lung diseases (43) and are mechanistically linked to endothelial adhesion, vascular maturation, and remodeling (44), giving evidence of the profound impact of SARS-CoV-2 on endothelial dysfunction.

In line with the role of endothelial cells as cellular source for a plethora of regulators of both primary and secondary hemostasis, aggravated endothelial dysfunction in fatal COVID-19 was accompanied by extensive dysregulation of coagulation as reflected by augmented PAI-1 and tPA levels. Similarly, endothelial synthesis of nitric oxide and prostacyclin is impaired in COVID-19 which may promote platelet activation (18). However, coagulation factor FXIII, which is primarily derived from platelets and not endothelial cells, ran afoul with the aforementioned trend and decreased with poor outcome, potentially indicating impaired release from platelets. In addition, levels of circulating platelet microthrombi were not associated with outcome, further arguing against widespread prothrombotic platelet activation. Our results thus imply that secondary rather than primary hemostasis is prominently involved in COVID-19, and we speculate that secondary hemostasis is also causally involved in thromboembolic complications. Prothrombotic platelet function may be diminished in severe COVID-19, which would explain the lack of a beneficial effect of antiplatelet therapy.

In line with previous reports (15, 45, 46), tissue sections of postmortem COVID-19 lung biopsies revealed widespread pulmonary microthrombi, and the vast majority of pulmonary thrombi were fibrin-rich but platelet-low. These findings further corroborate the putative low prothrombotic capacity of platelets in fatal COVID-19 and may explain why antiplatelet therapy might not prevent thromboembolic complications in COVID-19. Accordingly, frequency of antiplatelet medication and aspirin use is not associated with the development of thrombosis or death (40).

Despite multiple reports on increased severity- and viral load-dependent basal activation of platelets in COVID-19 (19, 20, 22, 26, 47), our results suggest that platelet function may be impaired in fatal disease. Indeed, also others found that platelets of patients with COVID-19 can present with hyporesponsiveness toward *in vitro* stimulation which might be caused by platelet exhaustion (15, 20, 27, 28). In particular, platelet procoagulant response is reduced (25), suggesting a minor role of platelet activation for thrombotic events in COVID-19.

Although platelet dysfunction in fatally ill patients with COVID-19 thus does not seem to contribute to thromboembolic complications, platelet dysfunction may affect their hemostatic capacity which might be further compromised by antiplatelet medication. Indeed, about 2.3% of hospitalized patients with COVID-19 and 5.6% of severely ill patients experience major bleeding events (7), and in our prospective cohort, about 18% of deceased patients died from hemostatic complications such as (pulmonary) bleeding.

We therefore speculated that platelet dysfunction in COVID-19 may impair platelet spreading and lamellipodia formation which is a prerequisite for vascular surveillance, but not for thrombus formation (35, 48). However, comparison of platelet shape change did not reveal any differences between healthy donors and severely ill patients with COVID-19, indicating sustained inherent platelet functions for vascular integrity. Whereas our results could not provide evidence for compromised platelet-mediated vessel integrity in COVID-19, a potential influence of SARS-CoV-2 and disease severity on platelet function and their hemostatic capacity cannot be ruled out.

Indeed, we found that disease outcome was associated with alterations in plasma composition, conferring an outcome-dependent hyporesponsive platelet phenotype which was disproportionately exacerbated by antiplatelet medication. Whereas the underlying molecular mechanisms are still unclear, our data thus indicate that in severely ill patients with COVID-19, antiplatelet therapy could potentially aggravate platelet hyporesponsiveness and increase risk for hemostatic complications.

Notably, our study has some limitations; for one, our study explores a single-center cohort that was recruited before the emergence and spread of SARS-CoV-2 variants. Our retrospective cohort also displays a relevant proportion of missing values regarding laboratory data, and hence, conclusions about potential associations have to be considered with care. Due to the low sample size in our subcohorts, it was not possible to adjust for multiple factors (e.g., comorbidities), and therefore, the data are only age-adjusted. During the recruitment phase, prophylactic anticoagulation with heparin was included into standard COVID-19 treatment unless counter-indicated, and the low number of anticoagulant-free patients did not allow us to stratify patients for analysis. This would have been of particular interest in light of a recent study revealing that therapeutic-dose heparin was only beneficial over usual-care thromboprophylaxis in patients with moderate but not severe COVID-19 (9). Moreover, our prospective study cohort lacks matched healthy or non-COVID-19 controls that would enable us to discern general effects of COVID-19. However, given that 15% of uncomplicated patients were asymptomatic upon enrollment, our cohort covers the whole spectrum of disease severities and allows for outcome-associated deductions. Nevertheless, findings should be corroborated in an independent cohort.

Taken together, our study suggests that dysregulation of coagulation is more strongly involved in the development of thromboembolic complications in COVID-19 than platelet-mediated imbalances. Fatal disease was not associated with elevated circulating microthrombi, but contrarily with plasma-mediated platelet hyporesponsiveness. Platelet hyporesponsiveness was further observed in postmortem lung biopsies of patients with COVID-19, which showed fibrin(ogen)-rich and platelet-low thrombi. Accordingly, anticoagulation but not antiplatelet therapy ameliorated survival. Platelets are also important sentinels of vascular integrity and their lamellipodia formation is critical for preventing vascular leakage during inflammation. Despite the hyporesponsive nature of platelets in severe COVID-19, no difference in lamellipodia formation was observed, indicating sustained inherent platelet functions for vascular integrity. Still, plasma-mediated platelet hyporesponsiveness may impact platelet capacity to ensure vascular integrity, as hemostatic complications represent a common cause of death. Ultimately, our study raises awareness that administration of antiplatelet medication may be ill fitted to prevent thromboembolic events in severe COVID-19.

## Data Availability Statement

The original contributions presented in the study are included in the article/Supplementary Material, further inquiries can be directed to the corresponding author/s.

## Ethics Statement

The studies involving human participants were reviewed and approved by Ethics Committee of the Medical University of Vienna (EK1315/2020, EK1548/2020, and EK 20-079-VK) and the North East—Newcastle & North Tyneside 1 Research Ethics Committee (19/NE/0336). The patients/participants provided their written informed consent to participate in this study.

## Author Contributions

WS, BJ, JR, and AA contributed to study conception. WS, AP, DP, AS, LB, AK, and JR performed experiments. WS, AP, SH, HH, and LB analyzed data. JS, KK, JO, EP, ST MP, MT, CS, TS, MK, and AZ recruited and treated patients and provided data. WS and AA wrote the manuscript. AA supervised the study. All authors contributed to the article and approved the submitted version.

## Funding

AK is a Welcome Funded Sir Henry Fellow (218649/Z/19/Z). JR is a British Heart Foundation Intermediate Fellow (FS/IBSRF/20/25039). This work is part of the ACOVACT study of the Medical University of Vienna and is financially supported by grants of the Austrian National Bank to WS (OENB18450) and of the Austrian Federal Ministry of Education, Science and Research, the Medical-Scientific Fund of the Mayor of Vienna (COVID024) and the Austrian Science Fund (P32064 and P34783) to AA and (SFB54) to BJ.

## Conflict of Interest

The authors declare that the research was conducted in the absence of any commercial or financial relationships that could be construed as a potential conflict of interest.

## Publisher's Note

All claims expressed in this article are solely those of the authors and do not necessarily represent those of their affiliated organizations, or those of the publisher, the editors and the reviewers. Any product that may be evaluated in this article, or claim that may be made by its manufacturer, is not guaranteed or endorsed by the publisher.
