# Supplementary material for: Platelets and Antiplatelet Medication in COVID-19-Related Thrombotic Complications
Source: Front Cardiovasc Med. 2022 Jan 24;8:802566. doi: 10.3389/fcvm.2021.802566 (PMC8818754; doi:10.3389/fcvm.2021.802566)
Supplement: Supplementary file 1 [file Data_Sheet_1.PDF]

Supplementary Figures

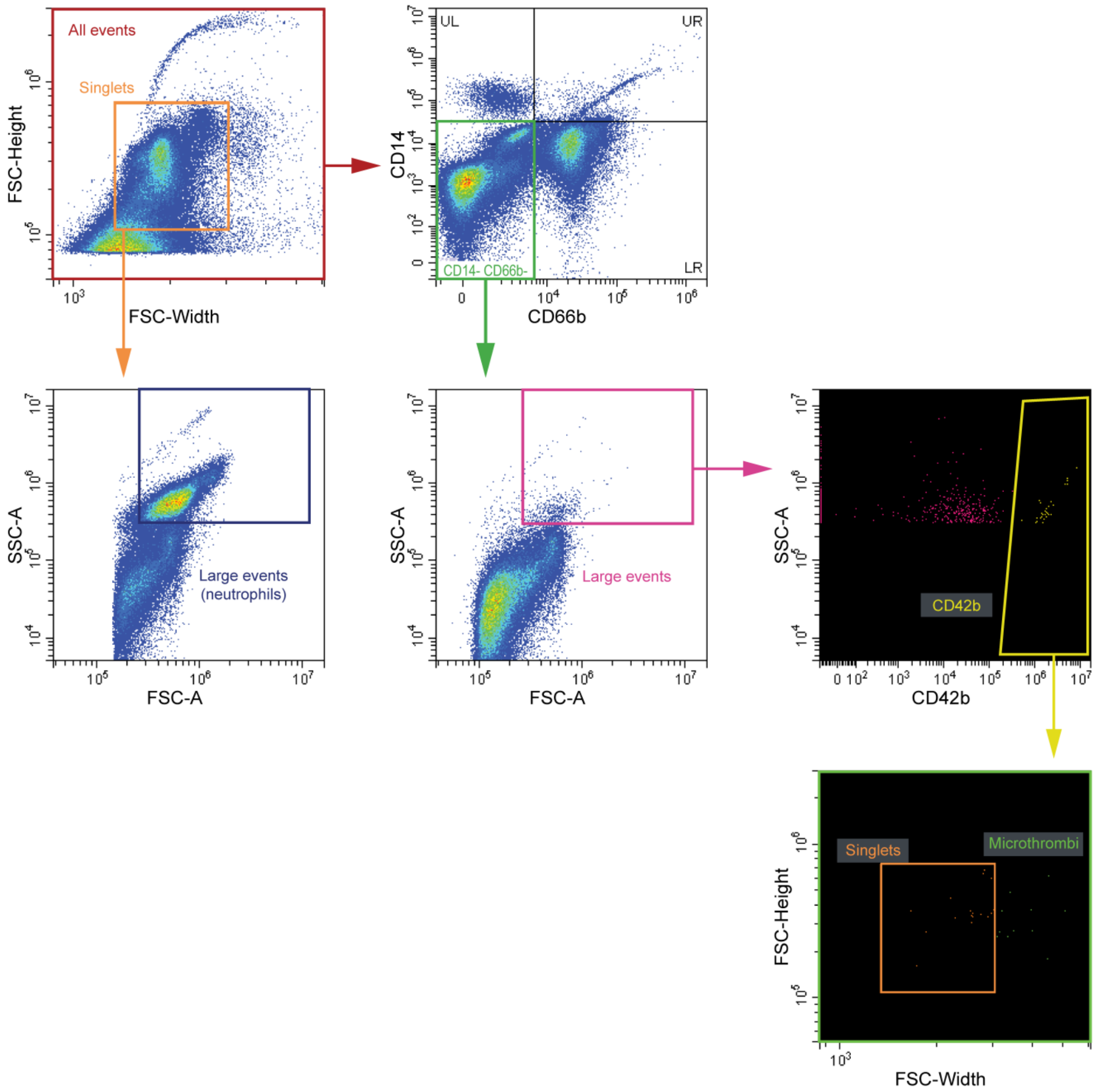

Supplement Figure 1: Gating strategy to determine circulating microthrombi. An FSC-Width/FSC-Height gate identifying singlets and an FSC-A/SSC-A gate encompassing large events such as neutrophils were drawn. All events were sub-gated to CD14- CD66b- events which were subsequently analyzed for size and CD42b. Microthrombi were determined as large CD14- CD66b- CD42b+ non-singlet events.

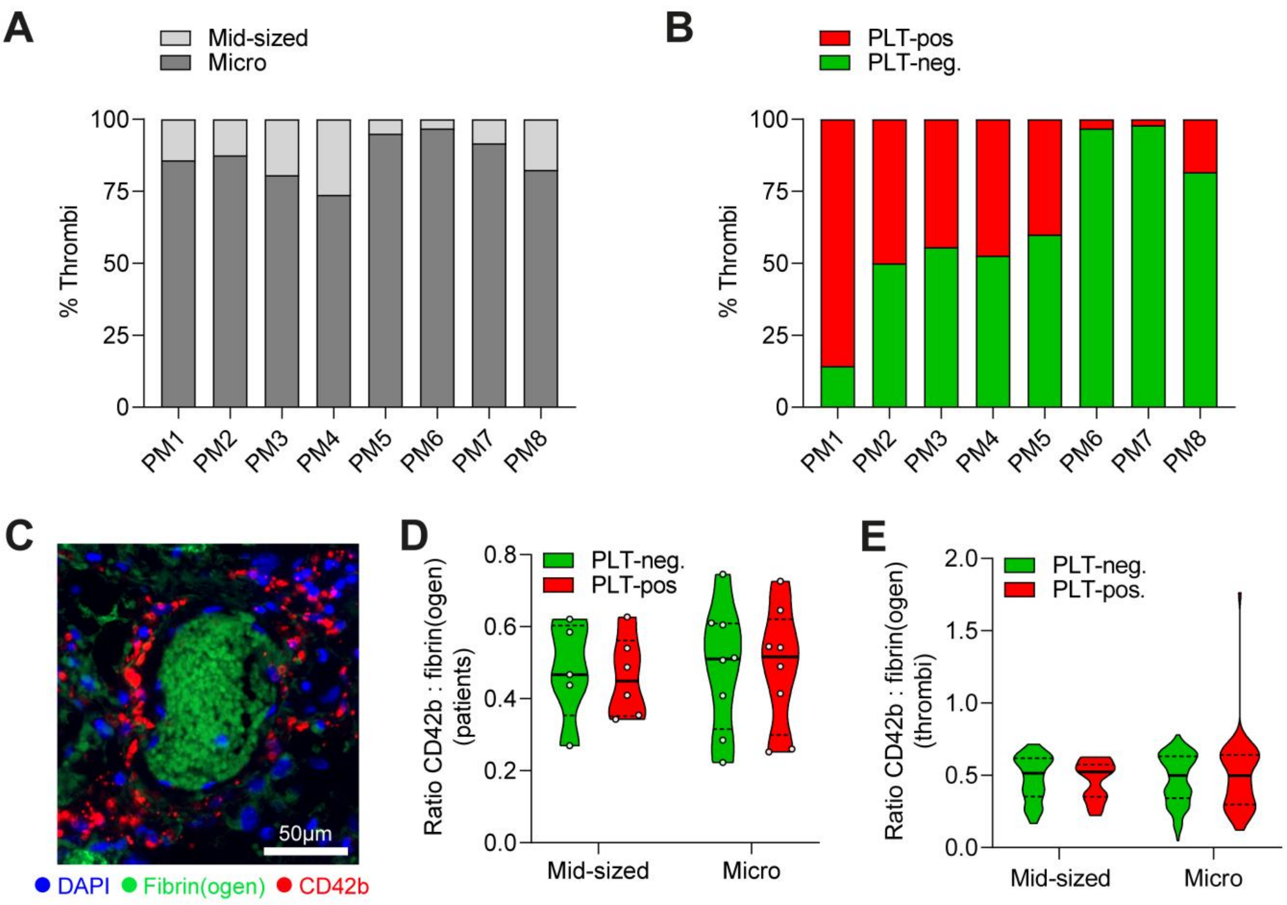

Supplement Figure 2: Characteristics of thrombi identified in post mortem lung sections of COVID-19 cases. Post mortem lung sections of 8 fatal COVID-19 cases were stained for CD42b, fibrin(ogen) and DAPI and thrombi identified by platelet and/or fibrin(ogen) accumulation. **(A-B)** Size and platelet-positivity of individual thrombi was evaluated and given as frequency (right panels) of identified thrombi. **(A)** Thrombi were classified according to their largest dimension into mid-sized (≥500µm) and microthrombi (<500µm). **(B)** Thrombi were classified into platelet-negative and platelet-positive. **(C)** Representative picture of ring-like platelet arrangements around some thrombi. **(D-E)** CD42b- and fibrin(ogen)-positive areas were quantified in pictures of individual thrombi. **(C)** Mean CD42b/fibrin(ogen) ratio per patient or **(D)** CD42b/fibrin(ogen) ratio of individual thrombi was compared between thrombi of different size and platelet positivity. n=8 patients.

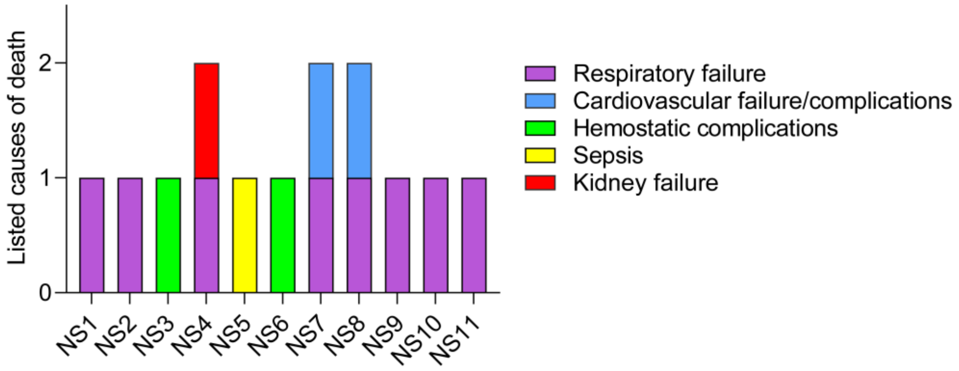

Supplement Figure 3: Listed causes of deaths (COD) among the prospective study cohort. Individual COD were extracted from medical files for all non-survivors within the prospective study cohort (NS1-11). In three cases multiple COD were listed. n=11 patients.
